# Cefepime-taniborbactam demonstrates potent *in vitro* activity vs *Enterobacterales* with *bla*_OXA-48_

**DOI:** 10.1128/spectrum.01144-24

**Published:** 2024-09-24

**Authors:** Maria F. Mojica, Elise T. Zeiser, Scott A. Becka, David A. Six, Greg Moeck, Krisztina M. Papp-Wallace

**Affiliations:** 1 Department of Molecular Biology and Microbiology, Case Western Reserve University, Cleveland, Ohio, USA; 2 Research Service, Veterans Affairs Northeast Ohio Healthcare System, Cleveland, Ohio, USA; 3 CASE-VA Center for Antimicrobial Resistance and Epidemiology, Cleveland, Ohio, USA; 4 Grupo de Resistencia Antimicrobiana y Epidemiología Hospitalaria, Universidad El Bosque, Bogotá, Colombia; 5 Venatorx Pharmaceuticals, Inc., Malvern, Pennsylvania, USA; 6 Department of Medicine, Case Western Reserve University, Cleveland, Ohio, USA; 7 Department of Biochemistry, Case Western Reserve University, Cleveland, Ohio, USA; Centre de Biologie Integrative, Toulouse, France

**Keywords:** β-lactamases, *Enterobacterales*, β-lactam, OXA-48, carbapenemase, β-lactamase inhibitor, cefepime-taniborbactam

## Abstract

Taniborbactam (formerly VNRX-5133) is a novel, investigational boronic acid β-lactamase inhibitor. The combination of cefepime (FEP) with taniborbactam is active against *Enterobacterales* carrying class A, B, C, and/or D enzymes. We assessed the activity of FEP-taniborbactam against *Enterobacterales* clinical strains carrying *bla*_OXA-48_ (*N* = 50, 100%), of which 78% harbored at least one extended-spectrum β-lactamase (ESBL). CLSI-based agar dilution susceptibility testing was conducted using FEP-taniborbactam and comparators FEP, meropenem-vaborbactam (MVB), and ceftazidime-avibactam (CZA). The addition of taniborbactam lowered FEP MICs to the provisionally susceptible range of ≤16 µg/mL; the MIC_90_ value decreased from ≥64 µg/mL for FEP to 4 µg/mL for FEP-taniborbactam. Notably, FEP-taniborbactam MIC_50_/MIC_90_ values (0.5/4 µg/mL) were lower than those for MVB (1/16 µg/mL) and comparable to those for CZA (0.5/1 µg/mL). Time-kill assays with *E. coli* clinical strains DOV (*bla*_OXA-48_, *bla*_CTX-M-15_, *bla*_TEM-1_, and *bla*_OXA-1_) and MLI (*bla*_OXA-48_, *bla*_VEB_, *bla*_TEM-1_, and *bla*_CMY-2_) revealed that FEP-taniborbactam at concentrations 1×, 2×, and 4× MIC displayed time-dependent reductions in the number of CFU/mL from 0 to 6 h, and at 4× MIC demonstrated bactericidal activity (3 log_10_ reduction in CFU/mL at 24 h). Therefore, taniborbactam in combination with FEP was highly active against this diverse panel of *Enterobacterales* with *bla*_OXA-48_ and represents a potential addition to our antibiotic arsenal.

**IMPORTANCE:**

OXA-48-like β-lactamases are class D carbapenemases widespread in *Klebsiella pneumoniae* and other *Enterobacterales* and are associated with carbapenem treatment failures. As up to 80% of OXA-48-like positive isolates coproduce extended-spectrum β-lactamases, a combination of β-lactams with broad-spectrum β-lactamase inhibitors is required to counteract all OXA-48-producing strains effectively. Herein, we evaluated the activity of cefepime-taniborbactam against 50 clinical strains producing OXA-48. We report that adding taniborbactam shifted the minimum inhibitory concentration (MIC) toward cefepime’s susceptible range, restoring its antimicrobial activity. Notably, cefepime-taniborbactam MIC_50_/MIC_90_ values (0.5/4 µg/mL) were comparable to ceftazidime-avibactam (0.5/1 µg/mL). Finally, time-kill assays revealed sustained bactericidal activity of cefepime-taniborbactam for up to 24 h. In conclusion, cefepime-taniborbactam will be a welcome addition to the antibiotic arsenal to combat *Enterobacterales* producing OXA-48.

## INTRODUCTION

β-lactams are the largest and most clinically prescribed class of antibiotics (1). β-lactams inhibit critical bacterial proteins involved in the synthesis of the cell wall known as penicillin-binding proteins, or PBPs, thus leading to bacterial cell death (2). The most common β-lactam resistance mechanism in Gram-negative bacteria is the production of β-lactamases, enzymes that hydrolyze the endocyclic amide bond of β-lactams. Based on structural and sequence characteristics, β-lactamases are grouped into four different Ambler classes A, B, C, or D. Class A, C, and D β-lactamases use serine as nucleophiles, while class B enzymes are Zn^2+^-dependent metallo-enzymes (3). Due to the ubiquity, functional and structural diversity, and ease of dissemination of β-lactamase encoding genes (*bla*) through different horizontal gene transfer strategies, both the Centers for Disease Control and Prevention and the World Health Organization have designated β-lactamase-producing Gram-negative bacteria as serious or critical threats (4–7). One of the most problematic and difficult-to-treat β-lactamase-producing Gram-negative pathogens is carbapenem-resistant *Enterobacterales* (CRE) that produce class D OXA-48-like carbapenemases (4).

OXA-48-like β-lactamases constitute carbapenem-hydrolyzing class D β-lactamases that are widespread in *Klebsiella pneumoniae* and other *Enterobacterales*. OXA-48, which was the first OXA-type carbapenemase isolated from enteric bacteria, is the most widespread member of this subgroup (8, 9). OXA-48 has a typical carbapenemase substrate profile with the highest catalytic efficiency for imipenem hydrolysis. However, due to their bulkier structures, OXA-48 activity against oxyimino-cephalosporins such as cefepime is very modest, and in the case of ceftazidime, undetectable (10–12). Therefore, in general, *Enterobacterales* producing only OXA-48-like enzymes are resistant to carbapenems but susceptible to ceftazidime and cefepime (13, 14). However, because most OXA-48-producing clinical isolates also carry extended-spectrum β-lactamases (ESBLs), these isolates can also display resistance to the aforementioned cephalosporins (15).

**Fig 1:**
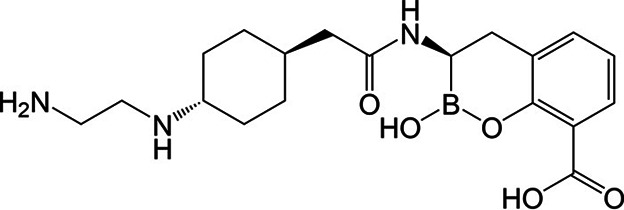
Chemical structure of taniborbactam.

To circumvent the effects of β-lactamases, β-lactamase inhibitors were developed and are given in combination with a partner β-lactam (16). Clinically available β-lactamase inhibitors possess one of three major chemical scaffolds: β-lactam-based (e.g., tazobactam), boronic acid-based (e.g., vaborbactam), or diazabicyclooctane-based (e.g., avibactam) (17). Taniborbactam (formerly VNRX-5133) is a boronic acid β-lactamase inhibitor that is in development in combination with cefepime (Fig. 1). Taniborbactam is unique compared to all clinically available β-lactamase inhibitors as it inhibits class A, B, C, and D β-lactamases (18, 19). Thus, cefepime-taniborbactam is active against *Enterobacterales* producing ESBLs, KPC, VIM, NDM, AmpCs, and OXA β-lactamases, but not strains carrying IMP metallo-β-lactamases (18, 20–25). Likewise, single amino acid variants of NDM-1 (NDM-9 and NDM-30) and VIM-1 (VIM-83) were recently shown to resist taniborbactam inhibition (26, 27). In *Enterobacterales*, impaired activity of cefepime-taniborbactam, although infrequent, has been reported due to co-production of carbapenemases with other β-lactamases (such as CTX-M-type ESBLs), carriage of PBP3 variants, and/or alteration or loss of porins, with multiple resistance mechanisms often present in the same organism (21, 28–30). Lastly, the combination demonstrated efficacy in various murine infection models caused by cephalosporin-resistant *K. pneumoniae* and carbapenem-resistant *Enterobacterales*, including metallo-β-lactamase producers (31–35). Furthermore, a phase 3 clinical trial comparing cefepime-taniborbactam to meropenem for the treatment of complicated urinary tract infections and acute pyelonephritis demonstrated that cefepime-taniborbactam was superior to MEM with a safety profile similar to that of meropenem (36).

In this study, the antimicrobial activity of cefepime-taniborbactam was compared to cefepime, meropenem-vaborbactam, and ceftazidime-avibactam against a challenge panel of clinical CRE producing OXA-48.

## RESULTS

### Bacterial strains

Our challenge panel of 50 clinical strains carrying *bla*_OXA-48_ included different species of *Enterobacterales* [*Escherichia coli*, *n* = 13; *Atlantibacter hermannii* (formerly *Escherichia hermannii*), *n* = 1; *Klebsiella oxytoca*, *n* = 2; *Klebsiella pneumoniae*, *n* = 30; and *Enterobacter cloacae, n =* 4]. Isolates were collected between 2007 and 2012 from France, Lebanon, Morocco, Algeria, Switzerland, the Sultanate of Oman, Egypt, Libya, the Netherlands, and Turkey. Additional information is given in the Materials and Methods section.

### Immunoblotting reveals that all 50 CRE produce the OXA-48 β-lactamase

**Fig 2:**
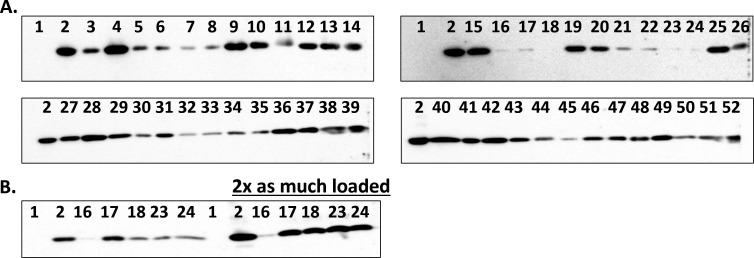
OXA-48 detection by western blot. (**A).** Immunoblotting was conducted using polyclonal anti-OXA-48 antibodies against the 50 CRE with bla_OXA-48_. *E. coli* DH10B pBC SK(+) empty and *E. coli* DH10B pBC SK(+) bla_OXA-48_ were used as controls and loaded in lanes labeled 1 and 2, respectively. (**B).** Immunoblotting was repeated for those isolates that had low levels of OXA-48 protein detected on the first blot using the same amount of protein loaded as in panel A and as well as 2× that vol (black line). Lanes 3–52, clinical strains loaded in the same order as presented in Table S2. Strains of special interest, *E. coli* MLI and *E. coli* DOV are shown in lanes 10 and 11, respectively.

To confirm the production of OXA-48 by the 50 CRE carrying *bla*_OXA-48_, immunoblotting was conducted with polyclonal anti-OXA-48 antibodies. All isolates produced detectable amounts of OXA-48 protein; however, as would be anticipated in a diverse collection of clinical isolates, there was variability of overall OXA-48 production levels (Fig. 2A). Strains *A. hermannii* DIA, *K. oxytoca* BOU, *K. oxytoca* IOZ, *K. pneumoniae* BAJ, and *K. pneumoniae* BEY produced lower amounts of OXA-48, and a second immunoblot was conducted loading 2× the amount of total protein (Fig. 2B). The OXA-48 from *A. hermannii* DIA at 2× was detectable, albeit the overall amount of OXA-48 produced was still lower than for all the other strains.

### Evaluation of agar dilution as a valid antimicrobial susceptibility testing method for cefepime-taniborbactam and comparators

Broth microdilution and agar dilution are standard methods approved by the Clinical Laboratories and Standards Institute (CLSI) to test the activity of antimicrobial agents. For a few agents, for example, polymyxin B, only broth microdilution is recommended, as some molecules do not diffuse efficiently in agar (37). For certain β-lactam/ β-lactamase combinations, such as ceftazidime-avibactam, it is clearly stated in the CLSI M100 that the MIC ranges for the QC strains were established using broth microdilution only and that equivalency data for agar dilution are not available. This is not the case for either cefepime/taniborbactam or meropenem/vaborbactam (37). Therefore, to validate the performance of agar dilution as a valid antimicrobial susceptibility testing method for ceftazidime-avibactam, we compared the MIC values for cefepime-taniborbactam, meropenem-vaborbactam, and ceftazidime-avibactam obtained by broth microdilution and agar dilution from CLSI-recommended control strains for β-lactam and β-lactamase inhibitor integrity, *K. pneumoniae* ATCC 700603 *bla*_SHV-18_, *K. pneumoniae* ATCC BAA-1705 *bla*_KPC_, and *E. coli* ATCC 25922. All controls were tested within the anticipated quality control ranges, where ranges were available (Table S1) (37). As the MIC values obtained by agar dilution for the quality control strains were within the published broth microdilution quality control ranges, agar dilution testing was confirmed to provide equivalent results to the reference broth microdilution method.

### Taniborbactam potentiates cefepime against *Enterobacterales* carrying *bla*_OXA-48_

**Fig 3:**
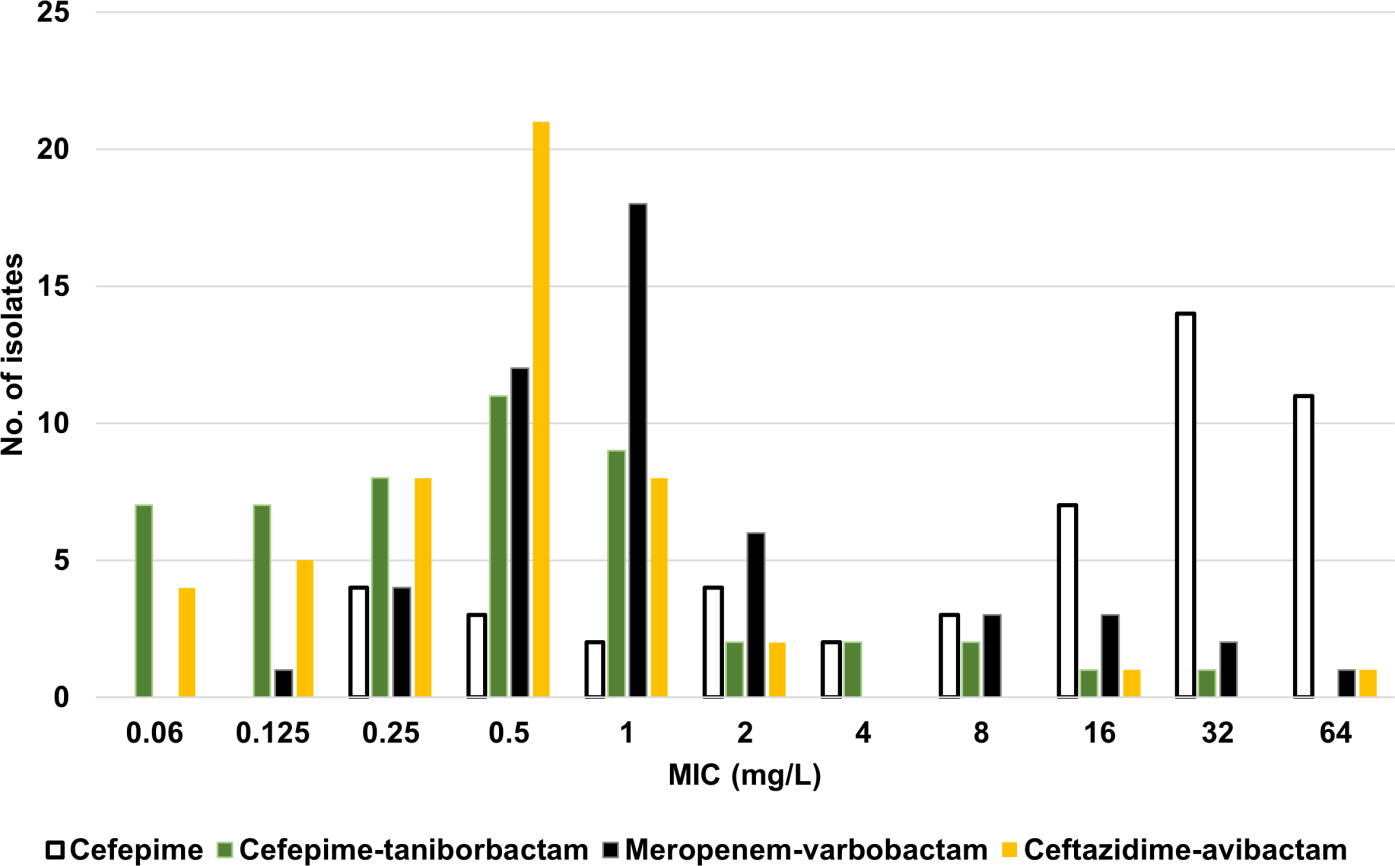
MIC distribution of cefepime-taniborbactam (green bars) compared to cefepime alone (white bars), meropenem-vaborbactam (black bars), and ceftazidime-avibactam (yellow bars) against 50 isolates of CRE carrying bla_OXA-48_.

Our challenge panel of clinical strains carried *bla*_OXA-48_ as well as other *bla* genes (*bla*_SHV_, *bla*_CTX-M_, *bla*_OXA_, *bla*_TEM_, *bla*_VEB_, and/or *bla*_CMY_). As seen in Table S2, 39/50 (78%) of strains carried at least one ESBL gene; *bla*_CTX-M-15_ was by far the most common, being present in 32/50 (64%) of the isolates. To assess the antimicrobial susceptibility of the isolates to cefepime, cefepime-taniborbactam, and comparators, we determined the MIC by the agar dilution methodology (Table 1). For comparative purposes only, FEP-taniborbactam MIC results were provisionally interpreted using cefepime breakpoints (37). In addition, the cumulative percentage of isolates inhibited at 16 µg/mL cefepime-taniborbactam was determined. An alternative cefepime-taniborbactam provisional susceptible breakpoint of ≤16 µg/mL is supported by *in vivo* efficacy data from neutropenic murine thigh, complicated urinary tract, and lung infection models (33, 34, 38) and data from safety and pharmacokinetics studies in human volunteers (21, 39, 40). As shown in Fig. 3 and Table 2, compared to cefepime alone, the addition of taniborbactam shifted the MIC values toward cefepime’s susceptible range (susceptible dose-dependent 4–8 μg/mL); the MIC_90_ value decreased from ≥64 μg/mL to 4 μg/mL. In addition, 98.0% and 96.0% of isolates were inhibited by cefepime-taniborbactam at ≤16 µg/mL and ≤8 µg/mL, compared to 50.0% and 36.0% for cefepime alone at ≤16 µg/mL and ≤8 µg/mL, respectively (Table 2). Cefepime-taniborbactam MIC_50_/MIC_90_ values (0.5/4 µg/mL) were lower than those of a clinically available comparator, meropenem-vaborbactam (1/16 µg/mL) (Table 1). For ceftazidime-avibactam, only two strains (4%) were resistant. For cefepime-taniborbactam, one strain had an MIC of 16 µg/mL [*K. pneumoniae* DIAR (*bla*_OXA-48_, *bla*_SHV-11_, *bla*_TEM-1_, *bla*_CTX-M-15_, *bla*_OXA-1_] and one strain had an MIC of 32 µg/mL [*K. pneumoniae* ELS (*bla*_OXA-48_, *bla*_SHV-11_, *bla*_TEM-1_, *bla*_CTX-M-15_, *bla*_OXA-1_] (Table S2). While *K. pneumoniae* DIAR was susceptible to ceftazidime-avibactam, *K. pneumoniae* ELS was cross-resistant to that combination. On the other hand, *E. coli* MLI (*bla*_OXA-48_, *bla*_VEB_, *bla*_TEM-1_, *bla*_CMY-2_) was resistant to ceftazidime-avibactam (MIC, 16 µg/mL for ceftazidime with avibactam fixed at 4 µg/mL), but provisionally susceptible to cefepime-taniborbactam (MIC, 2 µg/mL for cefepime with taniborbactam fixed at 4 µg/mL).

**TABLE 1:** Agar dilution MIC (µg/mL) against 50 isolates of CRE carrying *bla*_OXA-48_^*a*^^,^^*b*^

	FEP	FEP-TAN^*c*^	MVB^*c*^	CZA^*c*^
MIC_50_	16	0.5	1	0.5
MIC_90_	≥64	2	16	1
% Susceptible	26	88	82	96
% Susceptible dose-dependent (SDD) or intermediate	10	8	6	NA
% Resistant	64	4	12	4

^*a*^ Breakpoints for cefepime (susceptible (S) ≤ 2 µg/mL; susceptible dose-dependent (SDD) 4–8 µg/mL; resistant (R) ≥ 16 µg/mL) were used to assign phenotypes to cefepime and the combination with taniborbactam. The breakpoints for the other combinations are as follows: meropenem-vaborbactam (susceptible (S) ≤ 4 µg/mL; intermediate (I) = 8 µg/mL; resistant (R) ≥ 16 µg/mL), ceftazidime-avibactam (susceptible (S) ≤ 8 µg/mL; resistant (R) ≥ 16 µg/mL), NA, not applicable.

^*b*^ CZA, ceftazidime-avibactam; FEP, cefepime; MVB, meropenem-vaborbactam; TAN, taniborbactam.

^*c*^ Avibactam and taniborbactam were each tested at a fixed concentration of 4 µg/mL, while vaborbactam was tested at a fixed concentration of 8 µg/mL.

**TABLE 2:** MIC distributions depicting number of isolates at each MIC with cumulative percentage at MIC against 50 isolates of CRE carrying *bla*_OXA-48_^*a*^^,^^*b*^

	MIC (µg/mL)										
	≤0.06	0.125	0.25	0.5	1	2	4	8	16	32	≥64
Cefepime	0 (0%)	0 (0%)	4 (8%)	3 (14%)	2 (18%)	4 (26%)	2 (30%)	3 (36%)	7 (50%)	14 (78%)	**11** (**100%**)
Cefepime-taniborbactam^*c*^	7 (14%)	7 (28%)	8 (44%)	11 (66%)	9 (84%)	2 (88%)	**2** (**92%**)	2 (96%)	1 (98%)	1 (100%)	0 (100%)
Meropenem-vaborbactam^*d*^	0 (0%)	1 (2%)	4 (10%)	12 (34%)	18 (70%)	6 (82%)	0 (82%)	3 (88%)	**3** (**94%**)	2 (98%)	1 (100%)
Ceftazidime-avibactam^*c*^	4 (8%)	5 (18%)	8 (34%)	21 (76%)	**8** (**92%**)	2 (96%)	0 (96%)	0 (96%)	1 (98%)	0 (98%)	1 (100%)

^*a*^ For each MIC, the top number is the count of isolates with that MIC and the number in parentheses below it is the cumulative percentage. The zone shaded in gray represents the susceptible range. The MIC_90_ is shown in bold.

^*b*^ Gray shading represents the susceptible range.

^*c*^ Taniborbactam and avibactam were tested at a fixed concentration of 4 μg/mL.

^*d*^ Vaborbactam was tested at a fixed concentration of 8 μg/mL.

Across the combinations of β-lactam with β-lactamase inhibitor, the rank order of potency by MIC_90_ value against these CRE carrying *bla*_OXA-48_ was ceftazidime-avibactam > cefepime-taniborbactam > meropenem-vaborbactam. Across all tested compounds, the rank order of potency by MIC_90_ value against these CRE carrying *bla*_OXA-48_ was ceftazidime-avibactam > cefepime-taniborbactam > meropenem-vaborbactam > cefepime (Table 1; Fig. 3).

### Cefepime-taniborbactam demonstrates bactericidal activity against *E. coli* with *bla*_OXA-48_

**Fig 4:**
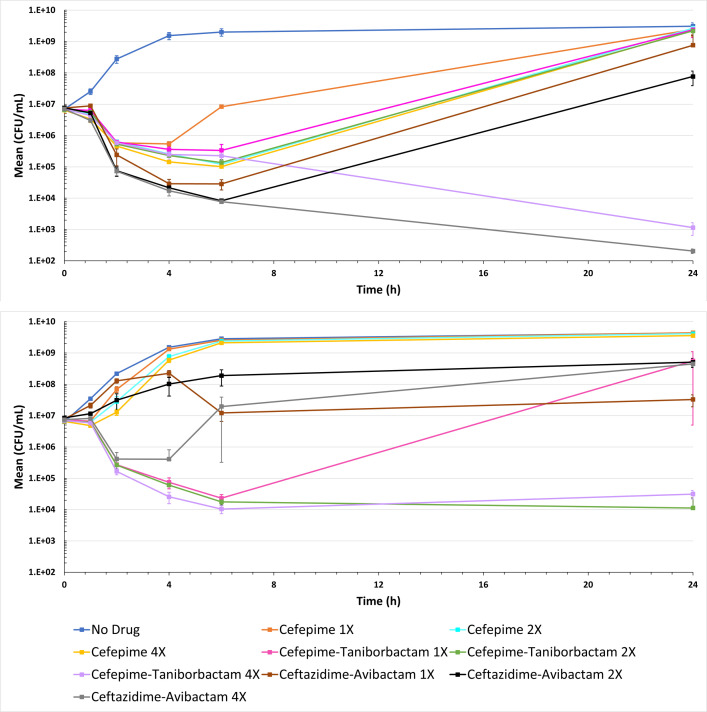
Time-kill experiment on *E. coli* DOV carrying bla_OXA-48_, bla_CTX-M-15_, bla_TEM-1_, bla_OXA-1_ (top) and *E. coli* MLI with bla_OXA-48_, bla_VEB_, bla_TEM-1_, bla_CMY-2_ (bottom). Bactericidal activity (≥ 3**-**log_10_ reduction at 24 h) was achieved against *E. coli* DOV by only ceftazidime-avibactam and cefepime-taniborbactam at 4× MIC and against *E. coli* MLI by only cefepime-taniborbactam at 2× and 4× MIC. The limit of detection in the assay was 100 CFU/mL.

Two clinical isolates, *E. coli* DOV (*bla*_OXA-48_, *bla*_CTX-M-15_, *bla*_TEM-1_, *bla*_OXA-1_) and *E. coli* MLI (*bla*_OXA-48_, *bla*_VEB_, *bla*_TEM-1_, *bla*_CMY-2_), were evaluated using time-kill kinetics at MIC multiples of 1×, 2×, and 4×. Each strain possessed the following MIC values: *E. coli* DOV, cefepime 16 µg/mL, cefepime-taniborbactam 0.25 µg/mL, and ceftazidime-avibactam 0.25 µg/mL; *E. coli* MLI, cefepime 128 µg/mL, cefepime-taniborbactam 2 µg/mL, and ceftazidime-avibactam 16 μg/mL (Table S2). Cefepime-taniborbactam demonstrated bactericidal activity against both tested strains of *E. coli*. For *E. coli* DOV only 4× cefepime-taniborbactam MIC sustained the bactericidal activity through 24 h (≥3-log_10_ decrease in the number of CFU/mL). Interestingly, against *E. coli* MLI, both 2× and 4× cefepime-taniborbactam MIC multiples maintained bactericidal activity through 24 h. Similarly, for *E. coli* DOV, a 2-log_10_ reduction in the number of CFU/mL was observed at 6 h while a 3-log_10_ reduction in the number of CFU/mL was observed for *E. coli* MLI in the same period (Fig. 4). As a direct comparator, ceftazidime-avibactam time-kill analyses were performed in parallel. For *E. coli* DOV only 4 × MIC achieved 3-log_10_ reduction in the number of CFU/mL at 6 h and bactericidal activity through 24 h. For *E. coli* MLI only a 2-log_10_ reduction in the number of CFU/mL was observed at 6 h with 4× MIC of ceftazidime-avibactam (Fig. 4).

## DISCUSSION

Over the past two decades, OXA-48-like enzymes have disseminated to become one of the most prevalent enterobacterial carbapenemases in Europe, Northern Africa, and the Middle East (41, 42). Despite the fact that OXA-48-like enzymes often cause only low-level *in vitro* resistance to carbapenems, they are associated with carbapenem treatment failures (13). Conversely, as ceftazidime, cefepime, and aztreonam evade hydrolysis by OXA-48-like enzymes (except in those with substitutions in residues located in or close to the β-5–β-6 loop, such as OXA-163) (43), these β-lactams could be thought as viable treatment options for infections caused by OXA-48-like-producing *Enterobacterales*. However, these drugs are labile to hydrolysis by ESBLs and, for ceftazidime and aztreonam, also by AmpCs. As we also showed here, up to 80% of OXA-48-positive isolates coproduce ESBLs (7, 61), thus, a broad-spectrum β-lactamase inhibitor in combination with either ceftazidime, cefepime, or aztreonam would be required to effectively counteract all OXA-48-producing strains (41).

The activity of a novel β-lactam-boronate-β-lactamase inhibitor combination, cefepime-taniborbactam, and comparator agents was evaluated against a challenge panel of clinical CRE isolates harboring *bla*_OXA-48_. As demonstrated *via* immunoblotting, all strains produced OXA-48 at different detectable levels. In agreement with the immunoblotting results, isolate *A. hermannii* DIA produced less OXA-48 and had the lowest meropenem-vaborbactam MIC value at 0.12 µg/mL compared to the other 49 strains (Fig. 2; Table S1). In most cases, however, the production level of OXA-48 was not directly related to the meropenem-vaborbactam MIC value. For example, strain *E. coli* BOU (*bla*_OXA-48_, *bla*_CTX-M-15_), which according to the immunoblotting produced the highest amount of OXA-48, displayed a meropenem-vaborbactam MIC value of 0.5 µg/mL, far from the >32 µg/mL values displayed by other strains with lower OXA-48 production levels. Nevertheless, regardless of the OXA-48 production levels, cefepime-taniborbactam demonstrated more potent *in vitro* activity compared to meropenem-vaborbactam, likely reflecting both taniborbactam inhibition of any OXA-48-mediated hydrolysis of cefepime, as well as inhibition of other class A and C β-lactamases capable of hydrolyzing cefepime. Vaborbactam possesses a more limited inhibitory profile that does not include OXA-48 carbapenemases (44); therefore, incomplete coverage of OXA-48-producing CRE by meropenem-vaborbactam is the reflection of the unprotected meropenem activity. Ceftazidime-avibactam and cefepime-taniborbactam showed equivalent *in vitro* activity against these isolates, with ceftazidime-avibactam inhibiting 96.0% of isolates at its breakpoint and cefepime-taniborbactam inhibiting 96.0% at ≤8 µg/mL and 98.0% (49/50 strains) at ≤16 µg/mL. These cefepime-taniborbactam susceptibility rates for OXA-48-producing *Enterobacterales* are comparable to other previously published reports (21, 30).

The MIC_50/90_ values for ceftazidime-avibactam (0.5/1 µg/mL) were similar to those for cefepime-taniborbactam (0.5/4 µg/mL). The contribution of β-lactamases other than OXA-48 to resistance phenotypes is hard to assess, as we did not determine their production level by immunoblotting as we did for OXA-48. Variation in β-lactamase levels in treated strains, reflecting basal and inducible expression, and/or differing lability of partner β-lactams to hydrolysis by the β-lactamase complement in each strain could explain how strains harboring the same β-lactamase genes displayed different susceptibilities to ceftazidime-avibactam and cefepime-taniborbactam. For example, *K. pneumoniae* DIAR, *K. pneumoniae* ELS, and *K. pneumoniae* DOV all carry *bla*_OXA-48_, *bla*_SHV-11_, *bla*_TEM-1_, *bla*_CTX-M-15_, and *bla*_OXA-1_, yet *K. pneumoniae* DOV was susceptible or provisionally susceptible to all β-lactam-β-lactamase inhibitor combinations, while the other two strains have different antibiograms altogether. Intrinsic differences in permeability and/or efflux in these clinical strains for cefepime and/or taniborbactam compared to ceftazidime and/or avibactam could explain the different potencies for these combinations. As was previously reported, the production levels and/or alterations of OmpK35/36 or OmpF/C in *K. pneumoniae* and *E. coli*, respectively, affect the entry of ceftazidime and/or avibactam somewhat differently than that of cefepime and/or taniborbactam (29, 30, 45–48). As a consequence, differences in the production level of OmpK35/36 or OmpF/C in these clinical strains may result in different susceptibility profiles.

Lastly, we performed time-kill kinetics tests at MIC multiples against two *E. coli s*trains, DOV and MLI. These strains were selected for two reasons. First, these strains represented different MIC ranges for ceftazidime-avibactam, which enabled a carefully controlled comparison of ceftazidime-avibactam time kills. This established the methodology of the time-kill that worked as expected against a well-characterized β-lactam/β-lactamase inhibitor combination in our hands, lending support to the parallel performance of the new investigational combination cefepime-taniborbactam. Second, these strains were selected for the collection of *bla* genes each harbored. The combination of *bla* genes present in the strain *E. coli* DOV (*bla*_OXA-48_, *bla*_CTX-M-15_, *bla*_TEM-1_, and *bla*_OXA-1_) was the most commonly found among all the isolates from our collection. Contrarily, *E. coli* MLI harbored a unique set of genes (*bla*_OXA-48_, *bla*_VEB_, *bla*_TEM-1_, and *bla*_CMY-2_). Results revealed that cefepime-taniborbactam was bactericidal up to 24 h, and are consistent with a previous report of cefepime-taniborbactam bactericidal activity against *E. coli* and *Pseudomonas aeruginosa* strains that produce metallo-β-lactamases (20). Importantly, the cefepime (≤8 µg/mL) and taniborbactam (4 µg/mL) concentrations represented in these time-kill studies are conservative in comparison to clinically achievable levels in the plasma of subjects receiving the 2.5 g (cefepime 2 g plus taniborbactam 0.5 g) dose every 8 h. Indeed, average plasma concentrations from the 2 g dose following IV infusion of cefepime (Maxipime) are 163.1 µg/mL at 0.5 h, 85.8 µg/mL at 1 h, 44.8 µg/mL at 2 h, 19.2 µg/mL, and 3.9 µg/mL at 8 h (49). Regarding taniborbactam, a Phase 1 pharmacokinetic study demonstrated that the mean taniborbactam maximum concentration in plasma is approximately 25 µg/mL following IV infusion and declined to approximately 10 µg/mL at 4 h (39).

Taken together, our results demonstrate the broad spectrum of activity of this novel combination. The *in vitro* activity of cefepime-taniborbactam against clinical CRE strains producing OXA-48 supports further clinical development of this combination.

## MATERIALS AND METHODS

### Antibiotics

Cefepime, ceftazidime, meropenem, and avibactam were purchased from Sigma; taniborbactam (lot#: CA 18–0590) and vaborbactam (lot#: RT-00097–129A) were provided by Venatorx.

### Quality controls strains

*K. pneumoniae* ATCC 700603 carrying *bla*_SHV-18_, *K. pneumoniae* ATCC BAA-1705 with *bla*_KPC_, and *E. coli* ATCC 25922 were used as controls for β-lactam and β-lactamase inhibitor integrity. *E. coli* ATCC 25922 constitutively produces low levels of chromosomally-encoded AmpC, a narrow-spectrum Ambler class C cephalosporinase (50).

### Bacterial strains

The 50 *Enterobacterales* clinical isolates carrying *bla*_OXA-48_ were purchased from Drs. Laurent Poirel and Patrice Nordmann at the University of Fribourg. The date of collection, location, and host sources were not recorded for all clinical strains at the time of isolation. However, of the data gathered on the clinical isolates used in this study, the isolates were obtained between the years of 2007–2012 from France, Lebanon, Morocco, Algeria, Switzerland, Sultanate of Oman, Egypt, Libya, the Netherlands, and Turkey. Host sources include sputum, urine, rectal swab, pus, blood, placenta, and bronchoalveolar lavage fluid. According to the CDC definition, these strains were deemed carbapenem-resistant *Enterobacterales* (CRE) as they tested resistant to at least one carbapenem and/or produced a carbapenemase (51).

For the immunoblotting control, the *bla*_OXA-48_ gene with the addition of a ribosomal binding sequence (TCTAGAAATAATTTTGTTTAACTTTAAGAAGGAGATATACAT) was synthesized and cloned into pBC SK (+) (Agilent) using XbaI and HindIII by Celtek Bioscience (Franklin, Tn). The pBC SK(+) with and without *bla*_OXA-48_ were electroporated into *Escherichia coli* DH10B (Invitrogen).

### Immunoblotting

The 50 *Enterobacterales* clinical isolates carrying *bla*_OXA-48_, as well as two controls *E. coli* pBC SK (+) empty and *E. coli* pBC SK (+) *bla*_OXA-48_, were grown in lysogeny broth (LB) to an optical density at 600 nm (OD_600nm_) of 0.6–0.8. One OD_600nm_ unit of cells was pelleted for 3 min at 10,000 rpm and the supernatant was discarded. The pellets were lysed in a 70 µL volume containing 50 mM tris(hydroxymethyl)aminomethane hydrochloride (Tris-Cl) at pH 7.4, 1 µM magnesium sulfate, 40 µg/mL lysozyme, and 1 µL benzonase nuclease. Lysates were pelleted for 3 min at 20,784 x *g* and 60 µL of the supernatant was mixed with 20 µL sodium-dodecyl-sulfate (SDS) loading dye containing 10% SDS, 0.05% bromophenol blue, 20% glycerol, 10 mM β-mercaptoethanol, and 0.2 M Tris-Cl, pH 6.8. The lysate mixture was boiled for 5 min at 99°C. Then, a 20 µL aliquot of each sample was loaded into each lane of the 10% SDS polyacrylamide gel; 2 × samples contained 40 µL of the sample. Five microliters of a protein molecular weight marker (Bio-Rad cat# 161375) were loaded onto one lane of each gel. The gels were run at 120 V until the dye front ran off the gel in 1 × Tris-glycine running buffer. Polyvinylidene difluoride membranes were activated by washing in methanol and running buffer at room temperature with shaking. Gels were transferred onto a polyvinylidene difluoride membrane at 80 amps for 3 h. The transferred membranes were blocked with 5% nonfat dry milk in 20 mM Tris-Cl (pH 7.4) with 150 mM NaCl (TBS) for 60 min at room temperature while shaking. The membranes were washed five times for 5 min each time with TBS + 0.05% Tween 20 (TBST) at room temperature with shaking and incubated overnight at 4°C with polyclonal anti-OXA-48 rabbit antibody at 1.5 µg/mL in 20 mL in TBS and 5% nonfat dry milk while shaking. The polyclonal anti-OXA-48 rabbit antibodies were raised by New England Peptide and isolated from serum with a protein G column (GE Healthcare) using the manufacturer’s instructions. The membranes were washed with TBST five times for 5 min each and then incubated with anti-rabbit protein G-horseradish peroxidase conjugate (Bio-Rad) for 60 min at room temperature with shaking. The blot was washed again with TBST five times for 5 min each at room temperature with shaking and then developed using the SuperSignal West Femto ECL developing kit (Thermo Scientific cat# 34096) according to the manufacturer’s instructions. The blots were imaged on an Azure 300 Imager for 15 s.

### *In vitro* susceptibility testing

MICs for the bacterial isolates were determined by the Mueller-Hinton (MH) agar dilution method with overnight cultures grown in MH broth and diluted in MH broth to 10^6^ colony forming units (CFUs)/mL (52). MIC measurements were performed using a Steers Replicator that delivered 10 µL of a diluted overnight culture containing 10^4^ CFU per spot. A second growth plate to detect contamination at the end of the assay was not used; instead, contamination was monitored by observing growth on each plate. Taniborbactam and avibactam were tested at 4 µg/mL in combination with increasing antibiotic concentrations of FEP and CAZ, respectively. Vaborbactam was fixed at 8 µg/mL with MEM at increasing concentrations. MIC results were interpreted using CLSI breakpoints, where available (37). For comparative purposes only, FEP-taniborbactam MIC results were provisionally interpreted using cefepime breakpoints (37). In addition, the cumulative percentage of isolates inhibited at 16 µg/mL FEP-taniborbactam was determined. An alternative FEP-taniborbactam provisional susceptible breakpoint of ≤16 µg/mL is supported by *in vivo* efficacy data from neutropenic murine thigh, complicated urinary tract, and lung infection models (33, 34, 38) and data from safety and pharmacokinetics studies in human volunteers (39, 40).

### Time-kill assay methods

Frozen glycerol stocks of *E. coli* DOV (*bla*_OXA-48_, *bla*_CTX-M-15_, *bla*_TEM-1_, and *bla*_OXA-1_) and *E. coli* MLI (*bla*_OXA-48_, *bla*_VEB_, *bla*_TEM-1_, and *bla*_CMY-2_) were streaked onto blood agar plates to obtain single colonies. Five colonies were resuspended into 10 mL of MH broth in 50 mL Falcon tubes and incubated at 37°C, 220 rpm until they reached an optical density at 600 nm (OD_600nm_) equivalent to a 0.5 McFarland standard. Cultures were then diluted in 10 mL of MH broth to reach the starting inoculum of 5 × 10^6^ CFU/mL in 50 mL conical (Falcon) tubes. The following compounds were added to *E. coli* DOV: cefepime (16, 32, 64 µg/mL), cefepime-taniborbactam (0.25, 0.5, 1 µg/mL of cefepime and taniborbactam at 4 µg/mL), and ceftazidime-avibactam (0.25, 0.5, 1 µg/mL of ceftazidime and avibactam at 4 µg/mL). The following compounds were added to *E. coli* MLI: cefepime (128, 256, and 512 µg/mL), cefepime-taniborbactam (2, 4, and 8 µg/mL of cefepime and taniborbactam at 4 µg/L), and ceftazidime-avibactam (16, 32, and 64 µg/mL of ceftazidime and avibactam at 4 µg/mL). The time that the drugs were added was designated as time zero. Cultures were incubated at 37°C, 220 rpm, and at 1, 2, 4, 6, and 24 h 100 µL was removed from each culture, serially diluted in MH broth, and 100 µL of each dilution was plated on MH agar. MH agar plates were incubated at 37°C for at least 18 h. Colonies were counted, and CFU/mL was determined. A ≥ 3-log_10_ decrease in the number of CFU/mL at 24 h was considered bactericidal. The deviations from the M26-A Methods for Determining Bactericidal Activity of Antimicrobial Agents Standard are the following: plastic tubes were used instead of glass, 100 µL of culture was diluted into 900 µL at the selected time points instead of 500 µL into 4.5 mL, dilutions were conducted in MH broth instead of 0.9% saline, and to avoid issues with colony formation, plates were streaked following the addition of each dilution instead of waiting 10–20 minutes prior to streaking (53).

## SUPPLEMENTAL MATERIAL

Table S1 spectrum.01144-24-s0001.docx Agar dilution susceptibility testing results for quality control strains.

Table S2 spectrum.01144-24-s0002.docx β-lactamase content and agar dilution MIC (μg/mL) against 50 isolates of CRE carrying blaOXA-48.

ASM does not own the copyrights to Supplemental Material that may be linked to, or accessed through, an article. The authors have granted ASM a non-exclusive, world-wide license to publish the Supplemental Material files. Please contact the corresponding author directly for reuse.
