# Supplementary material for: Cefepime-taniborbactam demonstrates potent in vitro activity vs Enterobacterales with blaOXA-48
Source: Microbiol Spectr. 2024 Sep 24;12(11):e01144-24. doi: 10.1128/spectrum.01144-24 (PMC11537129; doi:10.1128/spectrum.01144-24)
Supplement: Table S1 — Agar dilution susceptibility testing results for quality control strains. [file spectrum.01144-24-s0001.docx]

**Table S1.** Agar dilution susceptibility testing results for quality control strains.

| Strain | *bla* and porin genes | FEP | FEP-TAN* | MVB* | CZA* |
| --- | --- | --- | --- | --- | --- |
|  |  | MIC Ranges (µg/mL) | | | |
| *K. pneumoniae* ATCC 700603 | *bla*_SHV-18_  *bla*_OXA-2_  Mutations in  *ompK35* and *ompK37* | 0.5-2 | 0.25 | ≤0.06 | 0.5-2 |
| *K. pneumoniae* ATCC BAA-1705 | *bla*_KPC-2_  *bla*_TEM_  *bla*_SHV_ | 16 | 0.25 | ≤0.06 | 1-2 |
| *E. coli* ATCC 25922 |  | ≤0.06 | ≤0.06 | ≤0.06 | 0.12-0.25 |

*Avibactam and taniborbactam were each tested at a fixed concentration of 4 µg/mL, while vaborbactam was tested at a fixed concentration of 8 µg/mL. Abbreviations: FEP, cefepime; TAN, taniborbactam; MVB, meropenem-vaborbactam; CZA, ceftazidime-avibactam.
