# Supplementary material for: Cefepime-taniborbactam demonstrates potent in vitro activity vs Enterobacterales with blaOXA-48
Source: Microbiol Spectr. 2024 Sep 24;12(11):e01144-24. doi: 10.1128/spectrum.01144-24 (PMC11537129; doi:10.1128/spectrum.01144-24)
Supplement: Table S2 — β-lactamase content and agar dilution MIC (μg/mL) against 50 isolates of CRE carrying blaOXA-48. [file spectrum.01144-24-s0002.docx]

| **Table S2.** β-lactamase content and agar dilution MIC (μg/mL) against 50 isolates of CRE carrying *bla*_OXA-48_ | | | | | |
| --- | --- | --- | --- | --- | --- |
| **Strains** | ***bla* genes** | **FEP** | **FEP-TAN*** | **MVB*** | **CZA*** |
| *E. coli* Boui | *bla*_OXA-48_,  *bla*_CTX-M-15_,  *bla*_OXA-1_ | 16 | 0.12 | 0.25 | ≤0.06 |
| *E. coli* BOU | *bla*_OXA-48_,  *bl*a_CTX-M-15_ | 4 | ≤0.06 | 0.5 | 0.12 |
| *E. coli* CQ1 | *bla*_OXA-48_,  *bla*_CTX-M-24_ | 2 | ≤0.06 | 0.5 | ≤0.06 |
| *E. coli HAN* | *bla*_OXA-48_,  *bla*_CTX-M-15_ | 32 | 0.5 | 8 | 0.12 |
| *E. coli* LAL | *bla*_OXA-48_,  *bla*_CTX-M-15_,  *bla*_OXA-1_ | 0.25 | ≤0.06 | 0.25 | 0.12 |
| *E. coli* ESS | *bla*_OXA-48_,  *bla*_CTX-M -15_,  *bla*_TEM-1_ | 32 | ≤0.06 | 0.25 | 0.25 |
| *E. coli* KB-HAN | *bla*_OXA-48_,  *bla*_CTX-M -15_,  *bla*_TEM-1_ | 16 | ≤0.06 | 0.25 | ≤0.06 |
| *E. coli* MLI | *bla*_OXA-48_,  *bla*_VEB_,  *bla*_TEM-1_,  *bla*_CMY-2_ | >32 | 2 | 0.5 | 16 |
| *E. coli* DOV | *bla*_OXA-48_,  *bla*_CTX-M-15_,  *bla*_TEM-1_,  *bla*_OXA-1_ | 16 | 0.25 | 0.5 | 0.25 |
| *E. coli* 11663 | *bla*_OXA-48_,  *bla*_CTX-M-15_,  *bla*_TEM-1_,  *bla*_OXA-1_ | 32 | 1 | 32 | 0.25 |
| *E. coli* 11670 | *bla*_OXA-48_,  *bla*_CTX-M-15_,  *bla*_TEM-1_,  *bla*_OXA-1_ | 32 | 1 | 32 | 0.25 |
| *E. coli* KAD | *bla*_OXA-48_,  *bla*_TEM-1_ | 0.5 | 0.12 | 0.5 | 0.25 |
| *E. coli* 165 | *bla*_OXA-48_ | >32 | 8 | 2 | 2 |
| *A. hermannii* DIA | *bla*_OXA-48_ | >32 | 4 | 0.12 | 2 |
| *K. oxytoca* BOU | *bla*_OXA-48_ | 32 | 2 | 1 | 0.5 |
| *K. oxytoca* IOZ | *bla*_OXA-48_ | 32 | 0.5 | 0.5 | 1 |
| *K. pneumoniae* KID | *bla*_OXA-48_,  *bla*_SHV-28_ | 0.5 | 0.25 | 2 | 0.5 |
| *K. pneumoniae* DUW | *bla*_OXA-48_,  *bla*_SHV-28_,  *bla*_CTX-M-15_,  *bla*_TEM-1_ | >32 | 4 | 16 | 1 |
| *K. pneumoniae* VER | *bla*_OXA-48_,  *bla*_SHV-11_,  *bla*_CTX-M-15_,  *bla*_TEM-1_,  *bla*_OXA-9_ | 32 | 0.25 | 1 | 0.5 |
| *K. pneumoniae* BEN | *bla*_OXA-48_,  *bla*_SHV-28_,  *bla*_CTX-M-15_,  *bla*_TEM-1_ | >32 | 0.5 | 1 | 0.5 |
| *K. pneumoniae* BAJ | *bla*_OXA-48_,  *bla*_SHV-28_,  *bla*_CTX-M-15_,  *bla*_TEM-1_ | >32 | 0.5 | 1 | 0.5 |
| *K. pneumoniae* BEY | *bla*_OXA-48_,  *bla*_SHV-11_,  *bla*_TEM-1_,  *bla*_CTX-M-15_ | 32 | 0.5 | 1 | 0.5 |
| *K. pneumoniae* DAL | *bla*_OXA-48_,  *bla*_SHV-28_,  *bla*_CTX-M-15_,  *bla*_TEM-1_ | 16 | 0.5 | 1 | 0.5 |
| *K. pneumoniae* ELK | *bla*_OXA-48_,  *bla_S_*_HV-11_,  *bla*_TEM-1_,  *bla*_CTX-M-15_ | 32 | 0.5 | 1 | 0.5 |
| *K. pneumoniae* LOU | *bla*_OXA-48_,  *bla*_SHV-1_ | 0.25 | ≤0.06 | 1 | 0.25 |
| *K. pneumoniae* SIC | *bla*_OXA-48_,  *bla*_CTX-M-15_,  *bla*_SHV-28_ | 16 | ≤0.06 | 0.5 | ≤0.06 |
| *K. pneumoniae* SCO | *bla*_OXA-48_,  *bla*_SHV-11_ | 0.25 | 0.12 | 0.5 | 0.12 |
| *K. pneumoniae* DIAR | *bla*_OXA-48_,  *bla*_SHV-11_,  *bla*_TEM-1_,  *bla*_CTX-M-15_,  *bla*_OXA-1_ | >32 | 16 | >32 | 1 |
| *K. pneumoniae* DOV | *bla*_OXA-48_,  *bla*_SHV-11_,  *bla*_TEM-1_,  *bla*_CTX-M-15_,  *bla*_OXA-1_ | 0.5 | 0.12 | 1 | 0.5 |
| *K. pneumoniae* ELS | *bla*_OXA-48_,  *bla*_SHV-11_,  *bla*_TEM-1_,  *bla*_CTX-M-15_,  *bla*_OXA-1_ | >32 | 32 | 8 | >32 |
| *K. pneumoniae* ORS | *bla*_OXA-48_,  *bla*_SHV-28_,  *bla*_CTX-M-15_,  *bla*_TEM-1_ | 16 | 0.25 | 1 | 0.5 |
| *K. pneumoniae* CIR | *bla*_OXA-48_,  *bla*_SHV-70_,  *bla*_TEM-1_,  *bla*_CTX-M-15_ | 16 | 0.12 | 0.5 | 0.25 |
| *K. pneumoniae* NAJ301 | *bla*_OXA-48_,  *bla*_SHV-121_,  *bla*_CTX-M-15_,  *bla*_OXA-1_ | 8 | 0.25 | 0.5 | 0.5 |
| *K. pneumoniae* LIB | *bla*_OXA-48_ | 1 | 0.25 | 2 | 0.12 |
| *K. pneumoniae* ROU | *bla*_OXA-48_,  *bla*_CTX-M-15_ | 32 | 0.5 | 1 | 0.5 |
| *K. pneumoniae* PLE | *bla*_OXA-48_ | 2 | 1 | 8 | 0.5 |
| *K. pneumoniae* 16 | *bla*_OXA-48_ | >32 | 1 | 16 | 1 |
| *K. pneumoniae* RAM | *bla*_OXA-48_ | 2 | 0.5 | 1 | 0.5 |
| *K. pneumoniae* TIK | *bla*_OXA-48_ | 2 | 1 | 1 | 0.5 |
| *K. pneumoniae* Boui | *bla*_OXA-48_,  *bla*_CTX-M-15_ | 1 | 0.5 | 1 | 1 |
| *K. pneumoniae* HAN | *bla*_OXA-48_,  *bla*_CTX-M-15_ | 32 | 1 | 1 | 0.5 |
| *K. pneumoniae* ZED | *bla*_OXA-48_,  *bla*_CTX-M-15_,  *bla*_TEM-1_ | >32 | 1 | 2 | 1 |
| *K. pneumoniae* TUR1 | *bla*_OXA-48_,  *bla*_CTX-M-15_,  *bla*_TEM-1_ | >32 | 8 | 16 | 1 |
| *K. pneumoniae* AMS | *bla*_OXA-48_,  *bla*_CTX-M-15_,  *bla*_TEM-1_,  *bla*_OXA-1_ | 32 | 0.5 | 2 | 0.5 |
| *K. pneumoniae* BOU | *bla*_OXA-48_,  *bla*_CTX-M-15_ | 0.25 | 0.12 | 1 | 0.25 |
| *K. pneumoniae* EGY | *bla*_OXA-48_,  *bla*_CTX-M-15_ | 32 | 1 | 1 | 0.5 |
| *E. cloacae* MAR18 | *bla*_OXA-48_,  *bla*_CTX-M-9_,  *bla*_SHV-12_ | 8 | 0.25 | 1 | 0.5 |
| *E. cloacae* MAR19 | *bla*_OXA-48_,  *bla*_CTX-M-15_ | 32 | 1 | 0.5 | 0.5 |
| *E. cloacae* MAR20 | *bla*_OXA-48_,  *bla*_SHV-12_ | 8 | 0.25 | 2 | 1 |
| *E. cloacae* MAR17 | *bla*_OXA-48_,  *bla*_CTX-M-9_ | 4 | 0.12 | 0.5 | 0.5 |
| *Avibactam and taniborbactam were each tested at a fixed concentration of 4 μg/mL, while vaborbactam was tested at a fixed concentration of 8 μg/mL. Abbreviations: FEP, cefepime; TAN, taniborbactam; MVB, meropenem-vaborbactam; CZA, ceftazidime-avibactam | | | | | |
